# Integrated analysis and the identification of a circRNA-miRNA-mRNA network in the progression of abdominal aortic aneurysm

**DOI:** 10.7717/peerj.12682

**Published:** 2021-12-24

**Authors:** Ke Si, Da Lu, Jianbo Tian

**Affiliations:** 1 Department of Cardiovascular Surgery, The First Affiliated Hospital of Soochow University, Suzhou, Jiangsu, People’s Republic of China; 2 Department of Vascular Surgery, Shanghai General Hospital, Shanghai, People’s Republic of China; 3 Institute of Information Engineering, Chinese Academy of Sciences, Beijing, People’s Republic of China

**Keywords:** Abdominal aortic aneurysm, Differentially expressed genes, Micro RNAs, Circular RNAs

## Abstract

**Background:**

Abdominal aortic aneurysm (AAA) is a disease commonly seen in the elderly. The aneurysm diameter increases yearly, and the larger the AAA the higher the risk of rupture, increasing the risk of death. However, there are no current effective interventions in the early stages of AAA.

**Methods:**

Four gene expression profiling datasets, including 23 normal artery (NOR) tissue samples and 97 AAA tissue samples, were integrated in order to explore potential molecular biological targets for early intervention. After preprocessing, differentially expressed genes (DEGs) between AAA and NOR were identified using LIMMA package. Gene Ontology and Kyoto Encyclopedia of Genes and Genomes analysis were conducted using the DAVID database. The protein-protein interaction network was constructed and hub genes were identified using the STRING database and plugins in Cytoscape. A circular RNA (circRNA) profile of four NOR tissues *versus* four AAA tissues was then reanalyzed. A circRNA-miRNA-mRNA interaction network was constructed after predictions were made using the Targetscan and Circinteractome databases.

**Results:**

A total of 440 DEGs (263 up-regulated and 177 down-regulated) were identified in the AAA group, compared with the NOR group. The majority were associated with the extracellular matrix, tumor necrosis factor-α, and transforming growth factor-β. Ten hub gene-encoded proteins (namely IL6, RPS27A, JUN, UBC, UBA52, FOS, IL1B, MMP9, SPP1 and CCL2) coupled with a higher degree of connectivity hub were identified after protein‐protein interaction network analysis. Our results, in combination with the results of previous studies revealed that miR-635, miR-527, miR-520h, miR-938 and miR-518a-5p may be affected by circ_0005073 and impact the expression of hub genes such as CCL2, SPP1 and UBA52. The miR-1206 may also be affected by circ_0090069 and impact RPS27A expression.

**Conclusions:**

This circRNA-miRNA-mRNA network may perform critical roles in AAA and may be a novel target for early intervention.

## Introduction

A normal abdominal aorta has a diameter of approximately 30 mm. An abdominal aortic aneurysm (AAA) typically extends the aorta greater than or equal to 1.5 times this size. AAAs have an annual growth rate of 2.21 mm and few AAAs remain stable (Sweeting et al., 2012). Larger AAA carry a higher risk of rupture and the risk of death from a rupture can be as high as 81% (Guirguis-Blake et al., 2019). AAA is predominant in the elderly across the globe and is seen in 1.3% of men aged 65 years and 0.5% in women aged 70 years (Oliver-Williams et al., 2018; Svensjo, Bjorck & Wanhainen, 2013). The prevalence increases significantly among older people and may be as high as 7.2% in some areas (McCaul et al., 2016; Sampson et al., 2014a). AAA has a high mortality rate, causing an estimated 150,000 to 200,000 annual deaths worldwide (GBD 2013 Mortality and Causes of Death Collaborators, 2015; Sampson et al., 2014b). An abdominal aortic diameter of 55 mm is commonly regarded as the threshold for intervention. Early screening programs in many countries may introduce clinical interventions before the aneurysm reaches 55 mm. This may significantly increase treatment costs, but can improve patient survival (Tomee et al., 2017). In the United States alone, treatment for 30,000 to 40,000 AAA repair operations cost approximately $1 billion per year (Dua et al., 2014).

Open repair is a classic treatment for AAA but the development of endovascular repair has led to an increase in the number of patients receiving minimally invasive surgery. Clinical studies have revealed the benefits and deficits of both treatments (Trooboff et al., 2020). However, there is no clinical evidence to recommend surgical intervention in the early process of AAA expansion, and the results of existing drug trials are unsatisfactory. Propranolol, amlodipine and fenofibrate are the commonly used pharmaceuticals for treatment but none of them are beneficial in reducing the growth of small AAA (Bicknell et al., 2016; Pinchbeck et al., 2018; Propanolol Aneurysm Trial Investigators, 2002). The clinical trials using telmisartan, valsartan, ticagrelor and metformin for the treatment of AAA are still in progress (Golledge, 2019).

Previous studies have shown that there are a variety of genetic factors involved in AAA development. Patients who have a family history of AAA have twice the risk of developing it than those who do not (Larsson et al., 2009). A previous study of candidate genes revealed that more than 250 genes were AAA-related (Bradley et al., 2016). Genome-wide association studies have identified more strongly related genes, including *ERG*, *IL6R* and *LDLR* (Jones et al., 2017). With the development and application of high-throughput sequencing technology, a large number of differentially expressed genes (DEGs) have been detected in the expression profile of AAA (Biros et al., 2015; Gabel et al., 2017).

MicroRNA (miRNA) is a type of endogenous small, single-stranded non-coding RNA molecule, typically 18 to 24 nucleotides in length. Significant differences have been seen in miRNA expression levels in different tissues and during different developmental stages. miRNA can regulate gene expression by inhibiting mRNA translation or by reducing mRNA stability. MiRNA plays an important role in AAA. For example, miR-144-5p and miR-126 can regulate the formation and progress of AAA, and let-7f can be released into the blood as a new biomarker (Shen et al., 2020; Shi et al., 2020; Spear et al., 2019). The recent identification of dysregulated novel miRNA profiles has been studied in AAA plasma and tissues (Plana et al., 2020).

As a kind of non-coding RNA with a circular structure, circular RNA (circRNA) has a high degree of stability and conservation. CircRNA not only exists in tissues and organs, but is also widely distributed in peripheral blood. As a competitive endogenous RNA, circRNA regulates the expression of downstream target genes of miRNA by base complementary sponge adsorption miRNA (Gao et al., 2017). CircRNA plays a role in various cardiovascular diseases such as myocardial infarction, myocardial hypertrophy and heart failure (Han, Chao & Yao, 2018). However, only three circRNAs have been found to play a role in AAA (Yang et al., 2020b; Yue et al., 2020; Zhao, Chen & Jiang, 2020). For example, the CDR1as/miR 7/CKAP4 axis contributes to AAA pathogenesis by regulating the proliferation and apoptosis of primary vascular smooth muscle cells (VSMCs) (Zhao, Chen & Jiang, 2020). A recent study revealed 411 differentially expressed circRNAs by using microarrays to detect AAA’s non-coding RNA (Zhou et al., 2020a).

Microarray technology has recently been coupled with bioinformatics in the field of life science research (Chen et al., 2019). Microarray technology has identified large amounts of transcriptome profiling datasets. However, this data is limited by small sample sizes and a variety of platforms, which often lead to data deviation and inconsistency and prevent thorough analysis. Bioinformatics analysis can solve these problems by integrating the processing data through a variety of tools and facilitating the identification of the key genes and their regulators for disease progression.

Bioinformatics was used to reanalyze four gene expression profiles and one circRNA profile to identify the molecular mechanism behind the AAA progression (Biros et al., 2015; Gabel et al., 2017). Our analyses included DEGs screening, functional annotation, pathway enrichment and protein-protein interaction (PPI). Finally, a circRNA-miRNA-mRNA interaction network was constructed by combining previous studies with our predictions for micro RNAs (miRNAs) upstream of mRNAs and downstream of circRNAs. These analyses may provide new insights into AAA and facilitate the development of early therapeutic strategies.

## Materials and Methods

### Microarray datasets

**Figure 1:**
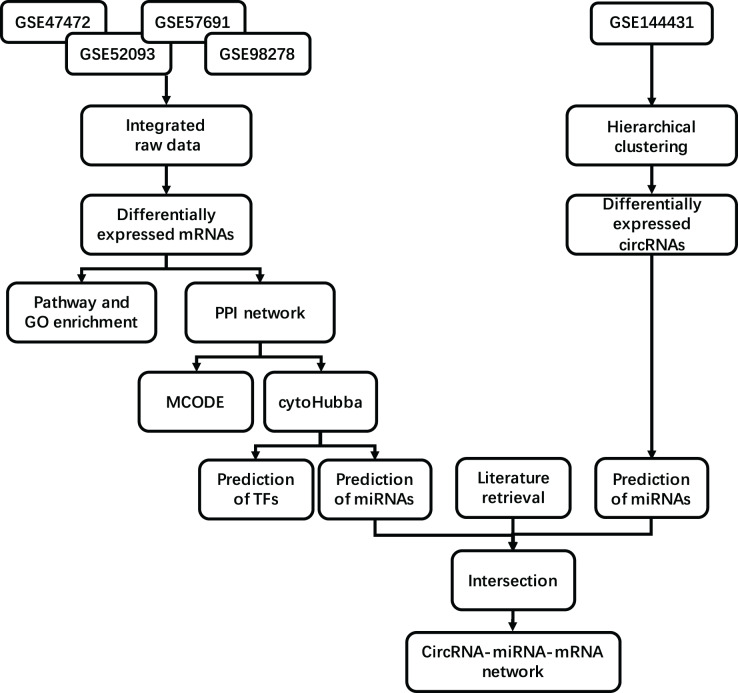
Study workflow. Flowchart for the integrated analysis of AAA microarray datasets from GEO and the studies obtained from PubMed.

The mesh term “abdominal aortic aneurysm” was retrieved from the Gene Expression Omnibus (GEO, https://www.ncbi.nlm.nih.gov/geo/) database. A considerable number of samples comparing AAA tissue and normal artery (NOR) tissue were found in the GPL10558 platform, including GSE47472 (published in 2013), GSE52093 (published in 2014), GSE57691 (published in 2015) and GSE98278 (published in 2018). We obtained 23 NOR tissue samples and 97 AAA tissue samples after integrating the data from all series. The NOR group incorporated arterial sample data from brain-dead patients described as normal or control, and the AAA group incorporated data from all samples described as AAA. All samples were detected using Illumina HumanHT-12 V4.0 Expression BeadChip on the GPL10558 platform. We included the circRNA profile GSE144431 from four NOR tissues *versus* four AAA tissues published in 2020. These eight samples were detected using ArrayStar Human CircRNA Microarray V2 on the GPL21825 platform. Details regarding the datasets are provided in Table 1. A flow diagram of the integrated analysis is shown in Fig. 1.

**Table 1:** Details from RNA microarray datasets used in this study.

Study type	Platform	Series	RNA	Samples (NOR/AAA)	Year	PMID
Gene expression profiling by array	GPL10558	GSE47472	mRNA	8/0	2013	–
Gene expression profiling by array	GPL10558	GSE52093	mRNA	5/0	2014	–
Gene expression profiling by array	GPL10558	GSE57691	mRNA	10/49	2015	25944698
Gene expression profiling by array	GPL10558	GSE98278	mRNA	0/48	2018	29191809
Non-coding RNA profiling by array	GPL21825	GSE144431	circRNA	4/4	2020	32039711

**Note:**

NOR, normal artery; AAA, abdominal aortic aneurysm.

### Integration of raw data

We included studies corresponding to these five datasets that were meant to address their respective concerns and that failed to integrate analysis. Data from 120 tissue samples of gene expression profiling was consolidated and raw data was downloaded from the GEO database. The data was subsequently processed by background correction to enhance the comparability of the raw data, normalize it, and prevent the batch effect (Fig. S1). The Illuminaio package (v0.28.0) in R software (v3.6.3) was used to process the raw data from all IDAT files to obtain the gene expression. CodesBinData was mapped to the Probe ID using the annotation file provided by the platform. The maximum values were computed as expression levels when multiple probes were matched to one gene symbol.

All data was subsequently imported and converted to a DGEList object using the edgeR package (v3.28.1). The raw library sizes were scaled and normalized using the function “calcNormFactors”. A model matrix was generated using combinations of two different groups, including NOR and AAA. The Limma package (v3.42.2) was used for modelling and fitting a linear model for each gene-given dataset. A pairwise comparison between groups was performed using a linear model and the eBayes function. The t-statistics, moderated F-statistic, p-value and log2 fold change (logFC) of differential expressions were calculated. To reduce the rate of false positives, the Benjamini–Hochberg method was applied to adjust the *p*-values. The thresholds for the DEGs were set as follows: up-regulated genes with logFC > 2 and adjusted-*p*-value < 0.05, down-regulated genes with logFC < −2 and adjusted-*p*-value < 0.05. The DEGs between the two groups were ultimately visualized in the heatmap. When processing the circRNA expression profile we used R software to perform the above-mentioned steps with the cut-off criteria set as |logFC| > 2 and *p*-value < 0.01.

### Gene ontology (GO) and Kyoto encyclopedia of genes and genomes (KEGG) analysis

The GO function annotation, founded by Gene Ontology Federation, may be used to explain the biological functions of genes and proteins using uniform terms. The KEGG pathway enrichment, established by Kanehisa Laboratory, integrates genomic, chemical and system functional information. GO functional annotation and KEGG pathway enrichment were conducted using the Database for Annotation, Visualization and Integrated Discovery (DAVID, http://david.ncifcrf.gov) with count > 2 and *p*-value < 0.05 thresholds (Kuleshov et al., 2016).

### The PPI network construction and hub genes identification

A list of DEG-encoded proteins was uploaded on the Search Tool for the Retrieval of Interacting Genes (STRING, http://string-db.org) online database to predict the PPI network (Szklarczyk et al., 2017). The interaction score > 0.4 and a maximum number of interactors = 0 were considered statistically significant and were visualized in Cytoscape software (v3.7.2). A new table including interactions was imported into Cytoscape with the Molecular Complex Detection (MCODE) plugin to screen the clustering modules of genes. The parameters set in MCODE were as follows: MCODE scores > 5, degree cut-off = 2, node score cut-off = 0.2, k-score = 2, and max depth = 100.

According to the algorithm, the degree is considered to be the number of protein interaction pairs for a certain protein. High-degree nodes seem to be essential for ensuring the stability of the whole network. Therefore, the degree of all nodes was calculated using the Cytoscape plugin cytoHubba at the same time (Chin et al., 2014). The DEGs with the 10 highest degree values were considered to be hub genes. The mean values of these hub gene expressions were shown in a boxplot.

### Prediction of miRNA and construction of the interaction network

The terms “miRNA” and “abdominal aortic aneurysm” were searched for, and all articles including experiments, clinical trials, sequencing studies and reviews were screened out from PubMed until April 30, 2021 (https://pubmed.ncbi.nlm.nih.gov/). All statistically significant miRNAs mentioned in these articles and their attachments were selected for deduplication. The Targetscan database was used to predict the upstream miRNAs of hub genes with all conserved sites or top 10 conserved sites as the result. The Circinteractome database was used to predict the downstream miRNAs of circRNAs with all the results included in the next analysis. The intersection of the above three was shown by the Venn diagram. The network of the intersection results was presented using Cytoscape.

## Results

### Identification of DEGs

**Figure 2:**
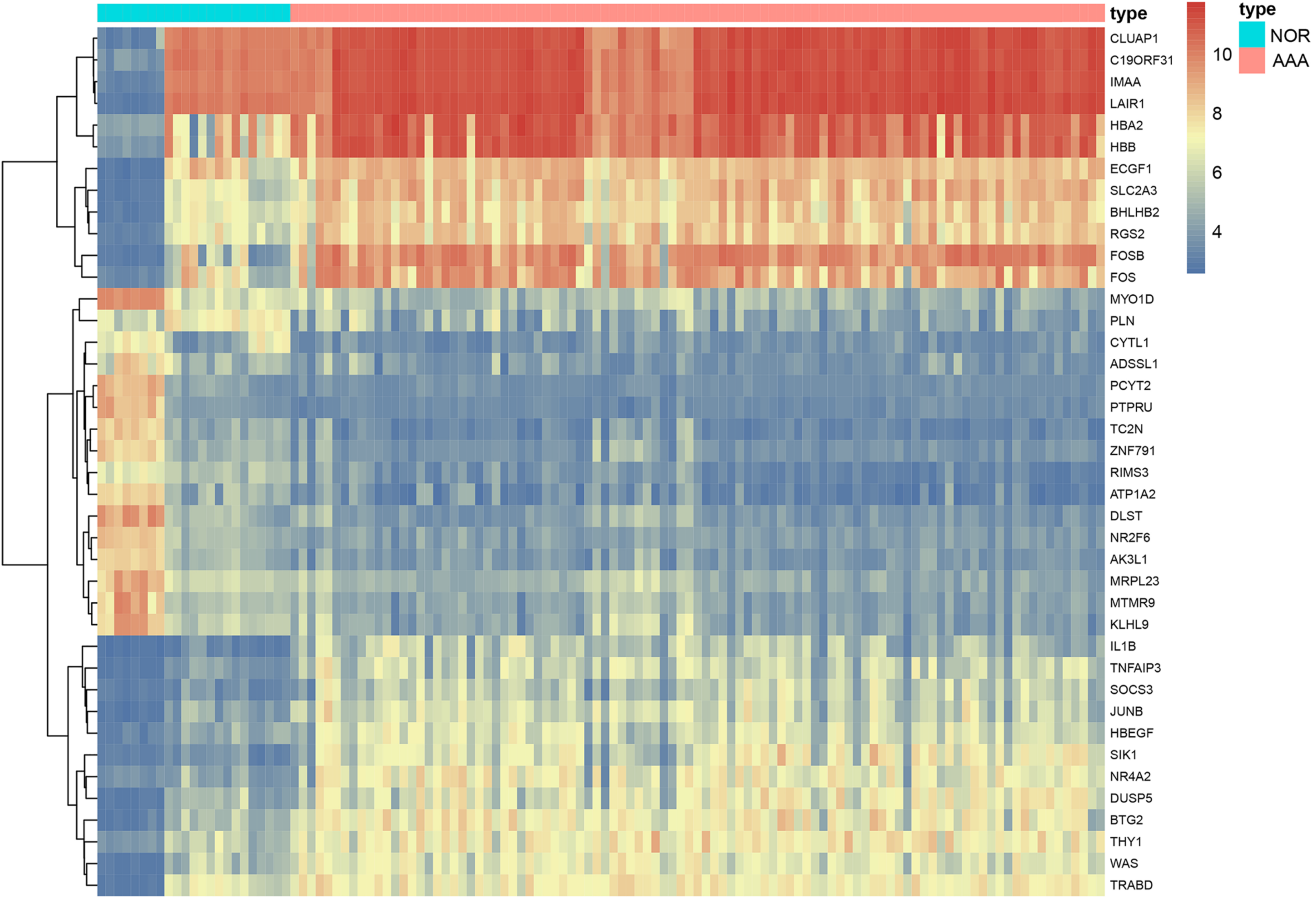
The heatmap of DEGs in AAA *vs* NOR. The heatmap depicts changes in gene expression profiles. The abscissa represents different samples and the ordinate represents DEGs. Maximum or complete linkage clustering was used for hierarchical clustering. AAA refers to abdominal aortic aneurysm group and NOR refers to the normal group.

We included 97 patients with AAA and 23 healthy controls from four gene expression profiling datasets, namely: GSE47472, GSE52093, GSE57691 and GSE98278. A total of 440 DEGs were identified in the AAA group following integrated bioinformatics analysis when compared with the NOR group (Table S1). The thresholds were set for adjusted-*p*-value < 0.05 and |logFC| ≥ 2. We found that 263 DEGs were up-regulated and 177 DEGs were down-regulated. The top five most significantly up-regulated in the AAA group sorted by logFC were *DNAJC19, STON1*, *OR13J1*, *SYNGR4* and *GPR115*. The top five most significantly down-regulated in the AAA group sorted by logFC were *FOS*, *LOC642113*, *HBB*, *HBA2* and *FOSB*. Significant differences are shown in Table 2 and Fig. 2.

**Table 2:** The top 40 significant DEGs sorted by adjusted-*p*-value.

Gene	logFC	AveExpr	t	*p*.Value	adj.*p*.Val	B
FOSB	−4.9900251	8.57710647	−17.020958	9.16E−34	8.70E−30	65.8112192
HBA2	−4.253595	9.7368147	−15.149615	1.30E−29	9.26E−26	56.5309914
RIMS3	2.25972762	4.00421194	15.0256972	2.48E−29	1.42E−25	55.9011126
HBB	−4.2674424	9.65862185	−13.70472	2.72E−26	9.71E−23	49.0794845
SLC2A3	−3.2569484	7.88289167	−12.068453	1.96E−22	3.29E−19	40.4025022
BTG2	−2.6963905	6.53922175	−11.822284	7.59E−22	1.03E−18	39.0812313
NR2F6	2.11615737	4.52920903	11.7057016	1.44E−21	1.64E−18	38.454477
CYTL1	2.12672937	4.08339053	11.2990126	1.35E−20	1.17E−17	36.2640087
SIK1	−3.0172671	5.89966192	−11.268946	1.59E−20	1.30E−17	36.1018608
ATP1A2	2.51773773	3.93264972	11.2145991	2.15E−20	1.70E−17	35.8087168
THY1	−2.2144457	6.76840417	−11.18231	2.57E-20	1.83E−17	35.634518
FOS	−3.5294184	8.1861442	−10.920224	1.09E−19	7.02E−17	34.2198877
NR4A2	−2.6982622	6.2183148	−10.894349	1.26E−19	7.80E−17	34.080178
MYO1D	2.58597366	5.54647738	10.6444795	5.00E−19	2.74E−16	32.7308529
TC2N	2.34950496	3.95105309	10.6053551	6.20E−19	3.21E−16	32.5195783
WAS	−2.2051087	6.23361893	−10.585215	6.93E−19	3.47E−16	32.4108227
BHLHB2	−2.6907251	7.58330317	−10.432374	1.61E−18	6.65E−16	31.5856122
MRPL23	2.14944996	5.22270307	10.3713955	2.25E−18	8.92E−16	31.2564679
AK3L1	2.05735854	4.36835923	10.3261382	2.89E−18	1.07E−15	31.0122233
ECGF1	−2.6837731	8.01503615	−9.9710018	2.04E−17	5.77E−15	29.097475
PLN	2.36220459	4.96147786	9.9562214	2.22E−17	6.20E−15	29.0178777
ADSSL1	2.01469743	4.29077794	9.898487	3.05E−17	7.97E−15	28.7070483
ZNF791	2.02279823	4.30599592	9.88020534	3.37E−17	8.73E−15	28.6086544
RGS2	−2.6286544	7.56777124	−9.8414479	4.17E−17	1.05E−14	28.4001087
DUSP5	−2.4907762	6.15291223	−9.731233	7.64E−17	1.77E−14	27.8074748
IL1B	−2.2362435	4.90235186	−9.6715405	1.06E−16	2.34E−14	27.4867779
JUNB	−2.1891372	5.60659029	−9.6650339	1.10E−16	2.40E−14	27.4518341
TNFAIP3	−2.2139601	5.38187463	−9.6640836	1.10E−16	2.40E−14	27.4467306
IMAA	−3.3093601	10.3636281	−9.5824058	1.73E−16	3.55E−14	27.008299
HBEGF	−2.1056812	5.72810859	−9.5819135	1.73E−16	3.55E−14	27.0056579
SOCS3	−2.1652887	5.15841563	−9.4569212	3.43E−16	6.32E−14	26.3355744
DLST	2.43852905	4.52505265	9.37093252	5.50E−16	9.38E−14	25.8752441
C19ORF31	−3.0301666	10.3847566	−9.3138426	7.51E−16	1.23E−13	25.5699395
CLUAP1	−3.2267642	10.3323884	−9.2967674	8.24E−16	1.32E−13	25.4786769
PCYT2	2.12743914	4.03690854	9.24988608	1.06E−15	1.66E−13	25.2282348
MTMR9	2.07088468	4.78877662	9.16976127	1.65E−15	2.41E−13	24.800653
TRABD	−2.0049887	6.84734965	−9.1045366	2.35E−15	3.24E−13	24.4530242
KLHL9	2.32006681	4.94195404	9.10451003	2.35E−15	3.24E−13	24.4528827
PTPRU	2.11344865	3.89592307	9.05054133	3.15E−15	4.11E−13	24.1655587
LAIR1	−3.3456012	10.3960057	−8.9884798	4.42E−15	5.48E−13	23.8355163

**Note:**

logFC, Log2-fold change between two experimental conditions; AveExpr, Average expression of gene symbol; t, Moderated t-statistic; *p*.Value, Raw *p*-value; adj.*p*.Val, *p*-value after adjustment for multiple testing; B, B-statistic or log-odds that the gene is differentially expressed.

### GO function annotation and KEGG pathway enrichment

**Figure 3:**
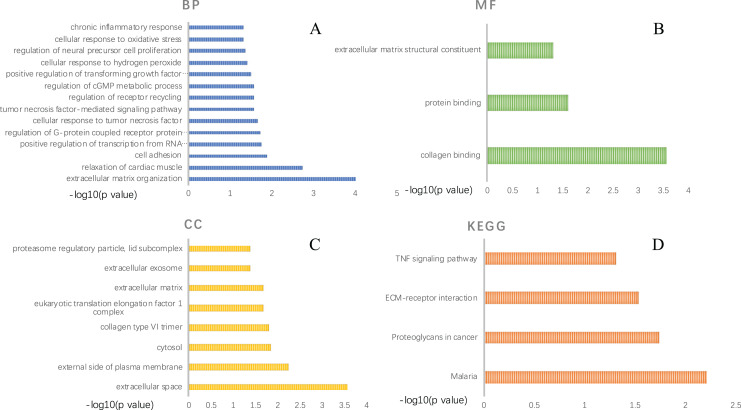
GO function annotation and KEGG pathway enrichment of DEGs selected by comparing AAA and NOR. GO function annotation includes three aspects: biological process (A), molecular function (B), and cellular component (C), KEGG pathway enrichment of DEGs (D). A *p*-value < 0.05 was considered significant.

GO functional analysis and KEGG pathway enrichment were performed using DAVID (Tables S2–S5). The GO functional analysis results classified the functional terms into three categories, namely: biological process (BP), molecular function (MF) and cellular component (CC). A critical value of *p* < 0.05 was selected. For BP, the top ten enriched DEGs ranked by *p*-value were: “response to extracellular matrix organization”, “relaxation of cardiac muscle”, “cell adhesion”, “positive regulation of transcription from RNA polymerase II promoter”, “regulation of G-protein coupled receptor protein signaling pathway”, “cellular response to tumor necrosis factor”, “tumor necrosis factor-mediated signaling pathway”, “regulation of receptor recycling”, “regulation of cGMP metabolic process” and “positive regulation of transforming growth factor beta1 production” (Figs. 3A). In the MF aspect, DEGs were enriched in “collagen binding”, “protein binding” and “extracellular matrix structural constituent”(Figs. 3B). As for CC, these DEGs were significantly related to “extracellular space”, “external side of plasma membrane”, “cytosol”, “collagen type VI trimer”, “eukaryotic translation elongation factor 1 complex”, “extracellular matrix”, “extracellular exosome” and “proteasome regulatory particle, lid subcomplex” (Fig. 3C). As presented in Fig. 3D, KEGG pathway enrichment results showed that the DEGs were significantly centralized at “Malaria”, “Proteoglycans in cancer”, “ECM-receptor interaction” and “TNF signaling pathway”.

### The PPI network construction and hub genes screening

**Figure 4:**
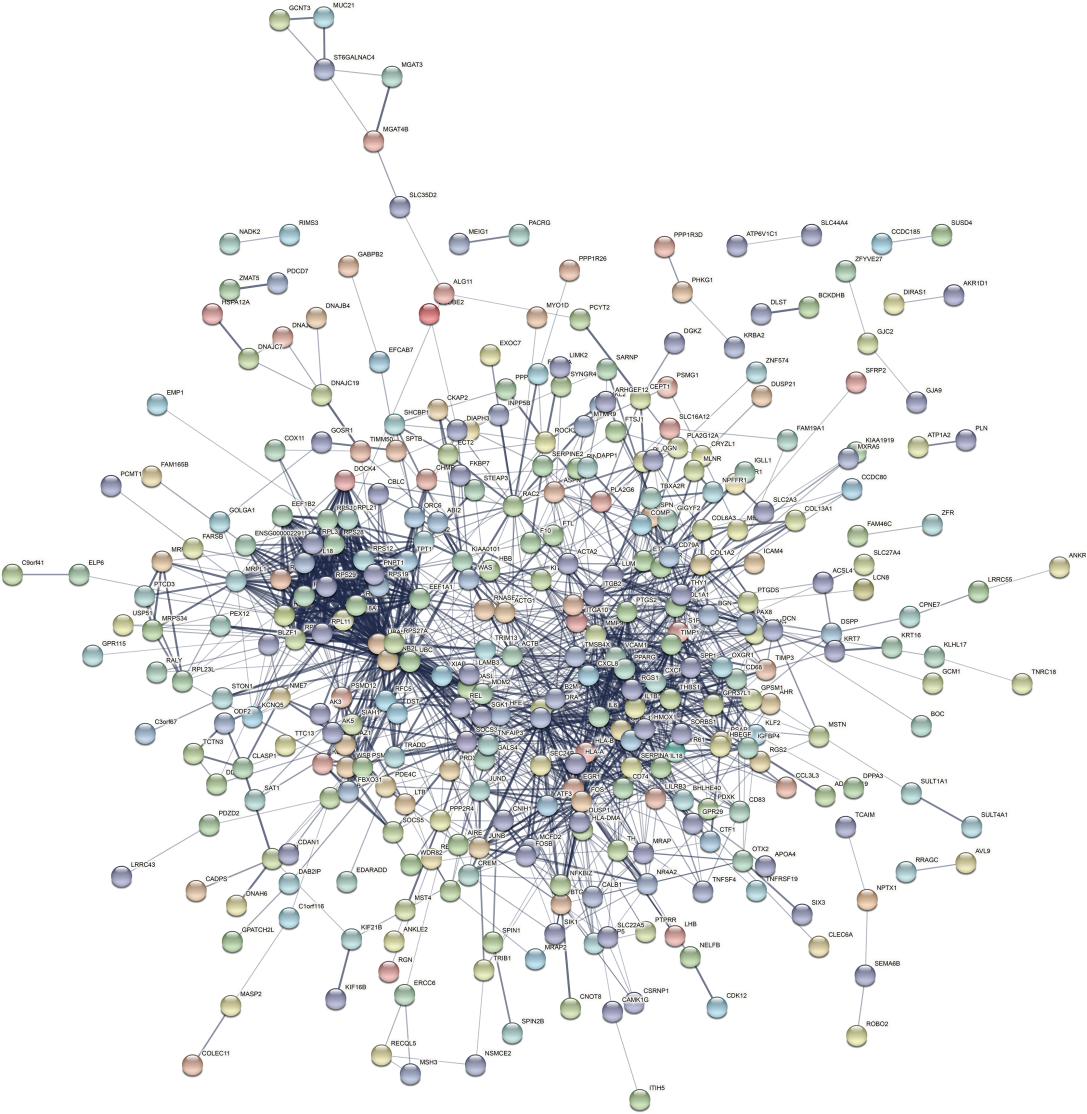
The PPI network constructed by DEGs with a linear relationship. The PPI network features 341 nodes and 985 edges and was constructed using Cytoscape.

**Figure 5:**
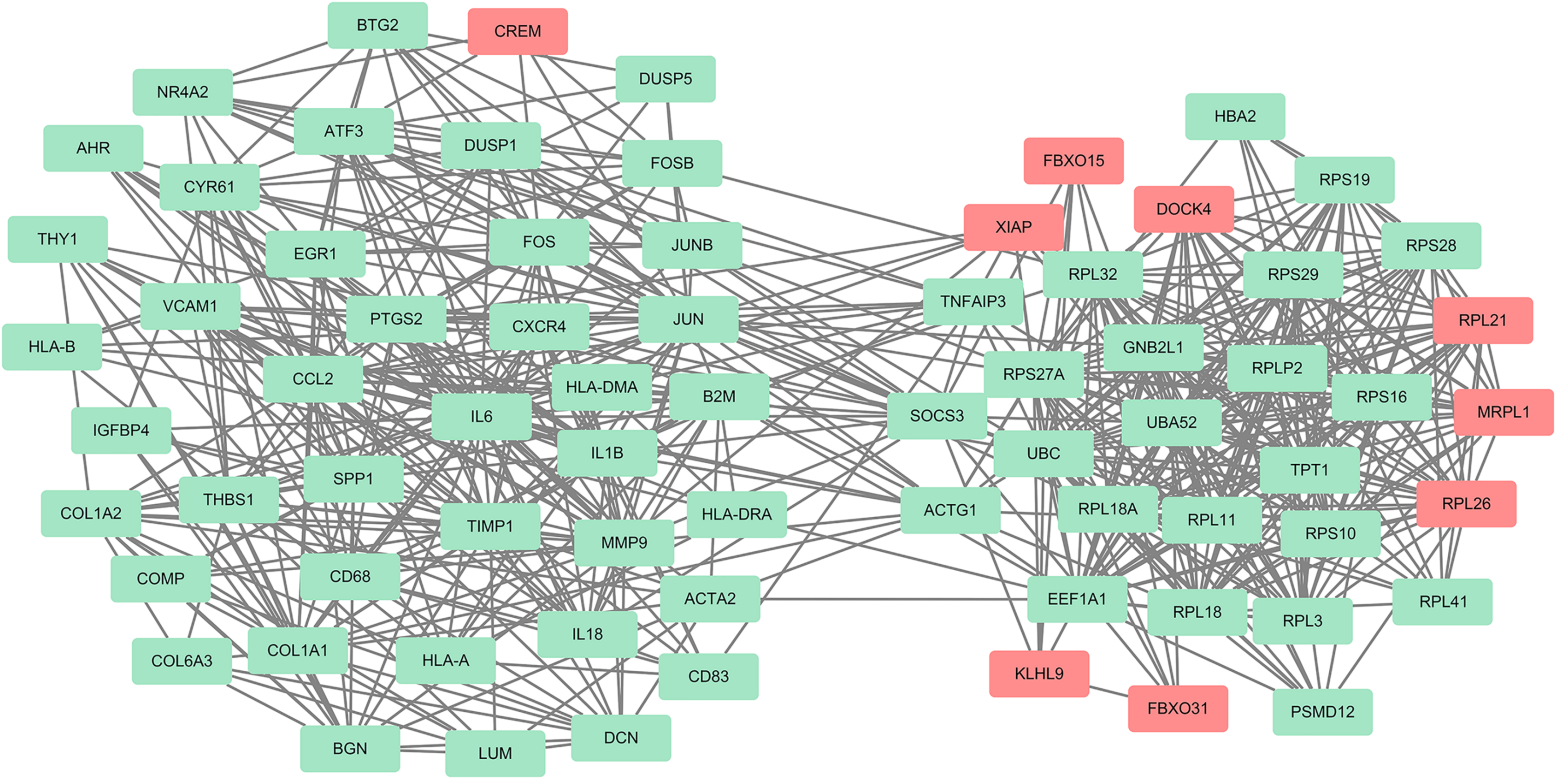
One module from the PPI network with tight clusters. One module with a score of 14.267 was analyzed using the plugin MCODE. Up-regulated genes are shown in red and down-regulated genes are shown in green.

**Figure 6:**
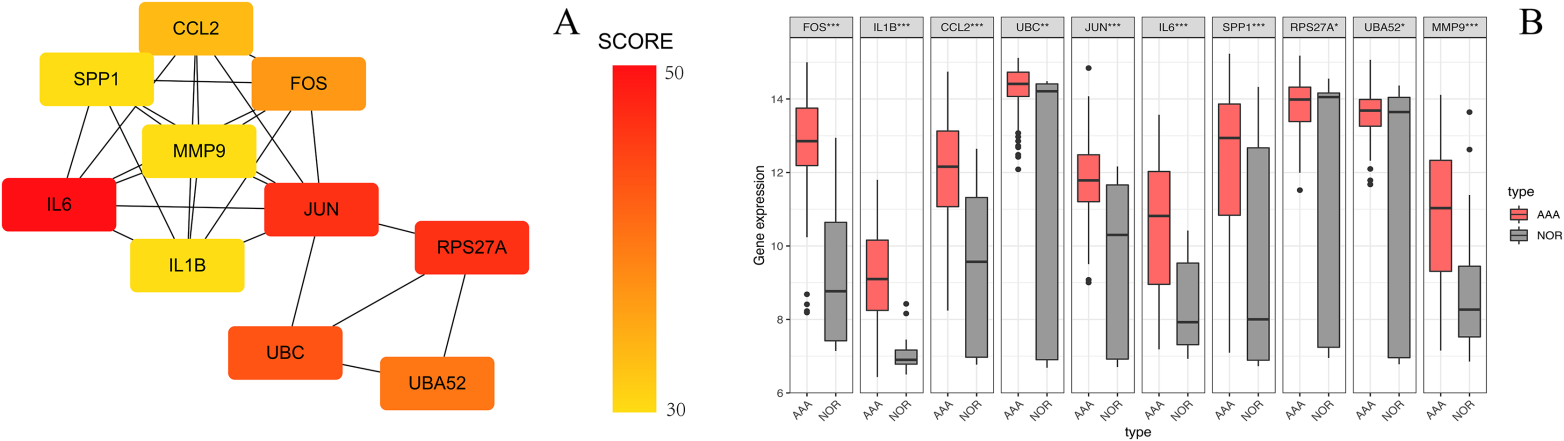
The top 10 hub genes in the PPI network. (A) The 10 identified hub genes screened by the plugin cytoHubba are displayed from a high degree value (red) to a low degree value (yellow). (B) The 10 box plots are based on the average expression value of 97 AAA samples marked in red and 23 NOR samples marked in gray. The upper and lower sides of the boxes represent the quartiles. The line inside the boxes represents the median. The end of the whiskers on boxes represents the maximum and minimum values. The dots at both ends represent the outliers.

The PPI network of the DEGs-encoded proteins was developed using the STRING online database and visualized with Cytoscape (Fig. 4). A total of 341 nodes and 985 edges were included in the PPI network. The most significant module was selected with score = 14.267 using the MCODE plugin to produce a better cluster of the function modules among the PPI network (Fig. 5). The module used nine up-regulated nodes and 63 down-regulated nodes. Subsequently, the top 10 hub genes-encoded proteins, including IL6, RPS27A, JUN, UBC, UBA52, FOS, IL1B, MMP9, SPP1 and CCL2, were identified using the cytoHubba plugin with a higher degree of connectivity hub. The boxplot displayed the mean values of these hub gene expressions (Figs. 6).

### Identification of differentially expressed circRNAs

**Figure 7:**
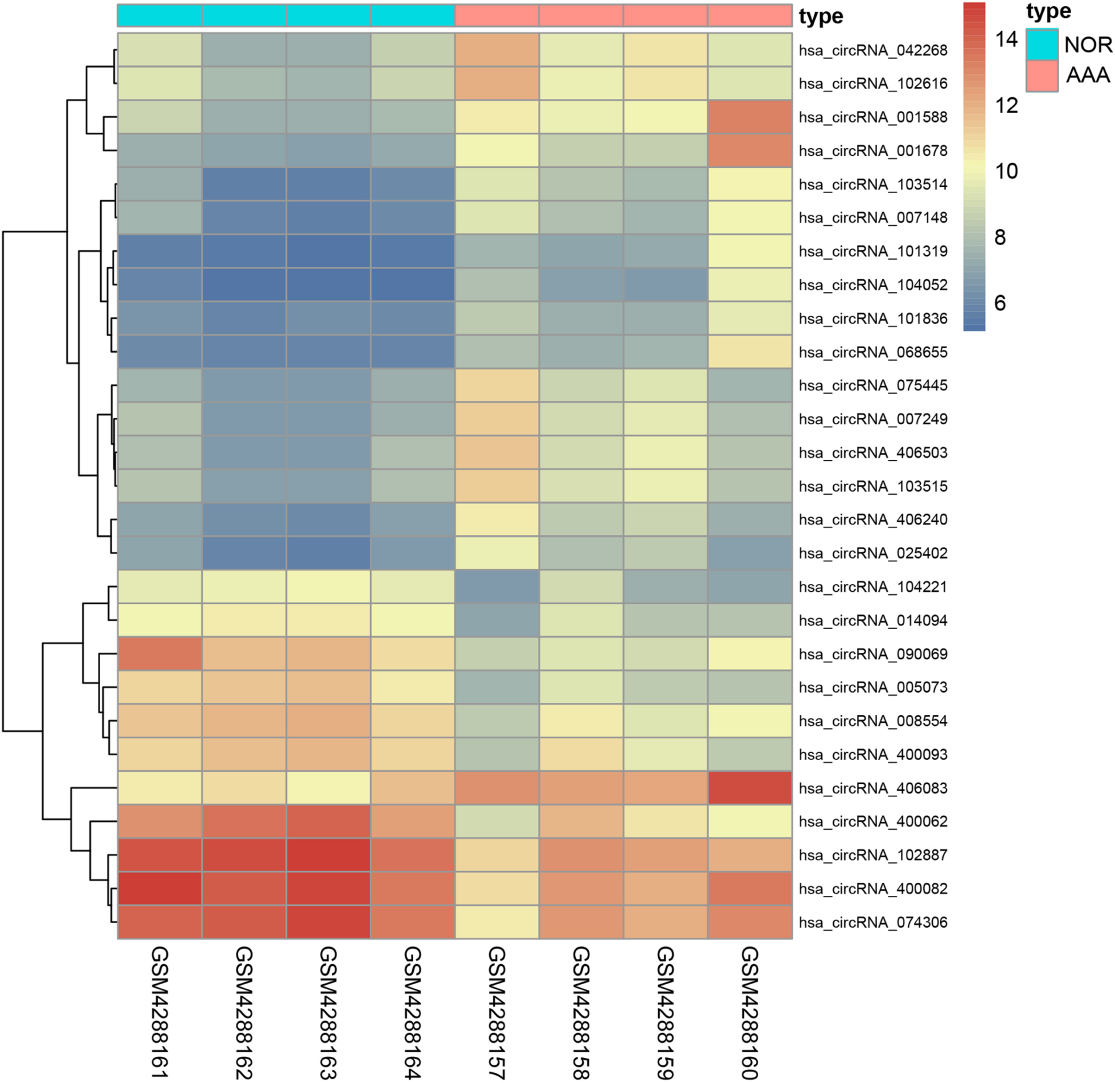
The heatmap of differentiall y expressed circRNAs. The abscissa represents different samples and the ordinate represents differentially expressed circRNAs. Maximum or complete linkage clustering was used for hierarchical clustering. As shown, AAA refers to the abdominal aortic aneurysm group and NOR refers to the normal group.

We identified 27 differentially expressed circRNAs, including 17 up-regulated and 10 down-regulated following GSE144431 dataset analysis (Table 3). The criteria for screening were set as follows: |logFC | > 2 and *p*-value < 0.01. The top three up-regulated and three down-regulated circRNAs ranked by logFC in the AAA group were: circRNA_001588, circRNA_001678, circRNA_400062, circRNA_103514, circRNA_005073, and circRNA_090069, respectively. A heatmap was drawn to reveal the gene expression between the samples (Fig. 7).

**Table 3:** The top 27 significantly differentially expressed circRNAs.

circRNA	logFC	AveExpr	t	*p*.Value	adj.*p*.Val	B
circRNA_005073	−2.6375668	9.74988938	−7.2384844	6.53E−05	0.09494774	2.05335296
circRNA_102887	−2.404863	13.242359	−5.6012191	0.00040919	0.09494774	0.47013378
circRNA_400062	−2.8588968	11.8039569	−5.1481194	0.00072278	0.09568055	−0.0437612
circRNA_104221	−2.2226655	8.66435825	−4.8664853	0.00104457	0.10348383	−0.3809727
circRNA_090069	−2.5822015	10.615549	−4.8152587	0.00111831	0.10348383	−0.4437881
circRNA_008554	−2.0236235	10.5831813	−4.7147018	0.00127996	0.10348383	−0.568419
circRNA_014094	−2.014015	9.19861175	−4.6883802	0.00132633	0.10348383	−0.6013317
circRNA_103514	2.73658825	7.54710413	4.67727952	0.00134643	0.10348383	−0.6152481
circRNA_101836	2.10932	7.15661625	4.47568033	0.0017748	0.10440485	−0.8716843
circRNA_406083	2.248287	11.9131803	4.29360549	0.0022894	0.1076509	−1.109257
circRNA_400093	−2.1661688	10.3354434	−4.1611209	0.00276374	0.10905833	−1.2856184
circRNA_007148	2.49781875	7.50083213	4.1609551	0.0027644	0.10905833	−1.2858409
circRNA_101319	2.50181225	6.71597763	4.09875878	0.00302254	0.11041958	−1.3696323
circRNA_001588	3.00762125	9.35924363	4.01909302	0.00339146	0.1113321	−1.4778695
circRNA_104052	2.4654355	6.5931525	3.92428556	0.0038942	0.1125509	−1.6079856
circRNA_406240	2.26076275	7.67991213	3.87314077	0.00419789	0.11429524	−1.6787547
circRNA_068655	2.47010825	7.14965288	3.80759339	0.00462442	0.11669599	−1.7700297
circRNA_400082	−2.130986	13.2986275	−3.7807697	0.00481205	0.11868744	−1.8075653
circRNA_074306	−2.104959	13.082301	−3.7607508	0.00495734	0.11983233	−1.8356469
circRNA_042268	2.2910685	9.28775625	3.65685106	0.00578991	0.12505368	−1.9823107
circRNA_406503	2.31075125	8.47736738	3.49928801	0.00734666	0.13041416	−2.2075164
circRNA_007249	2.22647675	8.36533363	3.43712984	0.0080772	0.13361283	−2.2972231
circRNA_103515	2.0994225	8.55234075	3.43539449	0.00809867	0.13361283	−2.2997342
circRNA_075445	2.137422	8.19193875	3.43032011	0.00816176	0.13361283	−2.3070789
circRNA_001678	2.95786325	8.57926363	3.4226246	0.00825844	0.13361283	−2.3182233
circRNA_025402	2.0367675	7.2966655	3.36124652	0.00907397	0.13600726	−2.4073508
circRNA_102616	2.02056775	9.45162013	3.31765822	0.00970416	0.13726971	−2.4708974

**Note:**

logFC, Log2-fold change between two experimental conditions; AveExpr, Average expression of gene symbol; t, Moderated t-statistic; *p*.Value, Raw *p*-value; adj.*p*.Val, *p*-value after adjustment for multiple testing; B, B-statistic or log-odds that the gene is differentially expressed.

### Prediction of miRNA and construction of the interaction network

**Figure 8:**
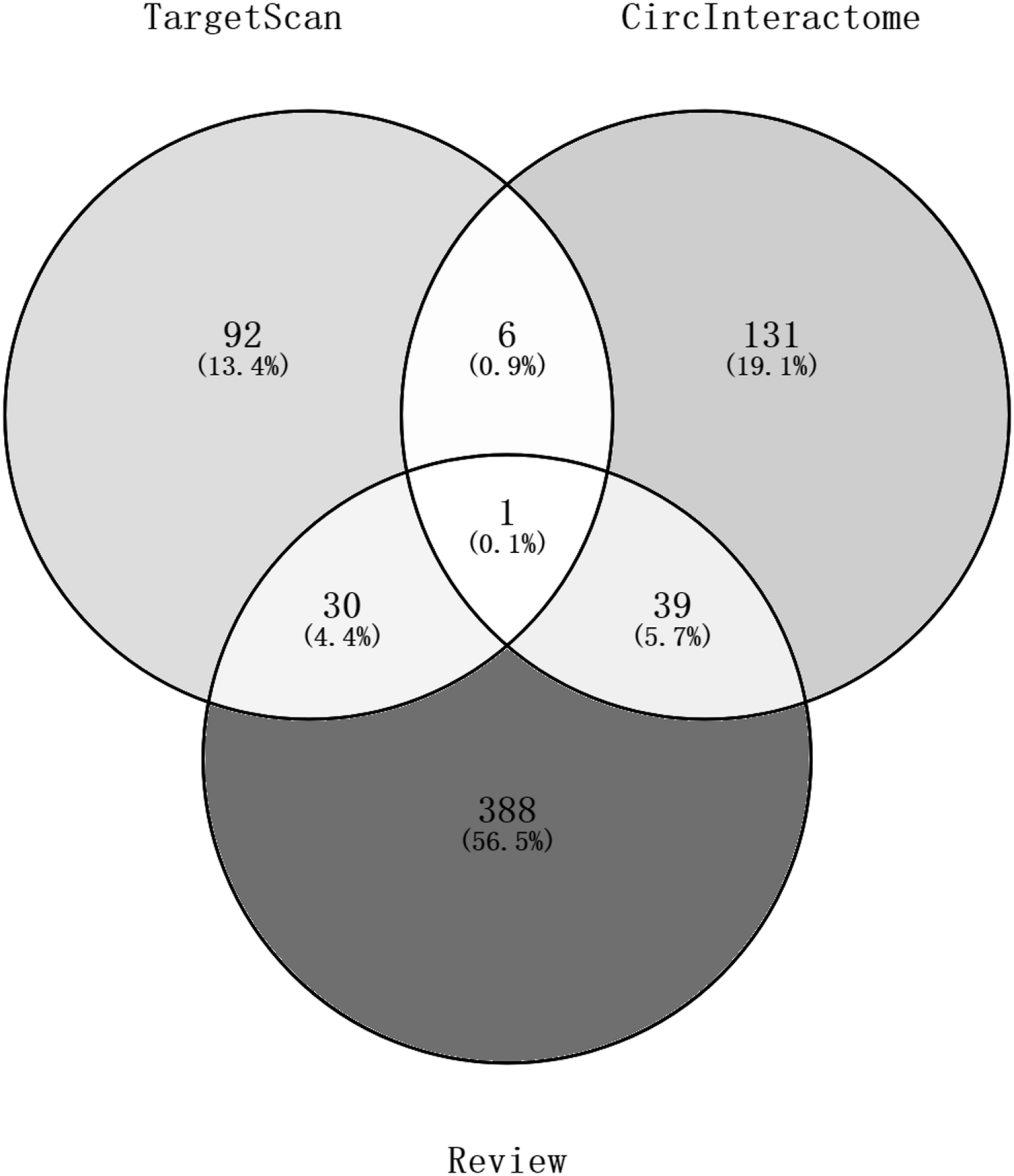
A Venn diagram of predictions from the targetscan database and Circinteractome database and alignments from previous studies in PubMed. The counts of miRNAs obtained from the overlap of circRNAs downstream prediction, mRNA upstream prediction and studies review are shown.

**Figure 9:**
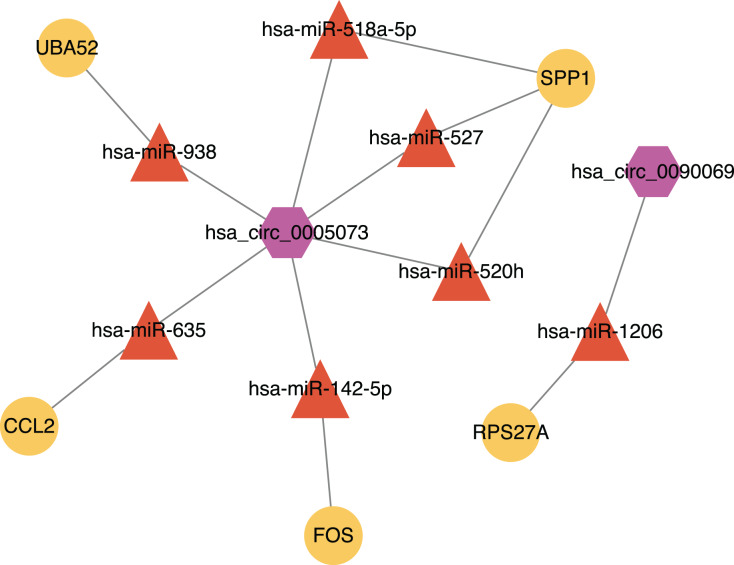
The circRNA-miRNA-mRNA network. Purple hexagons, circRNAs; orange triangles, miRNAs; yellow circles, mRNAs.

Ninety-two miRNAs were obtained using the Targetscan database to predict the miRNAs upstream of 10 hub genes. One hundred thirty-one miRNAs were obtained using the Circinteractome database to predict the miRNAs downstream of 6 circRNAs. Common to both predictions were seven miRNAs, including miR-142-5p, miR-635, miR-527, miR-520h, miR-938, miR-518a-5p and miR-1206. A total of 105 articles were retrieved as of April 31, 2021 following a review of the previous research. There were 388 statistically significant miRNAs mentioned in these articles after deduplication. The obtained miRNAs were intersected and displayed on a Venn diagram (Fig. 8). Finally, circRNAs, miRNAs and mRNAs with linear connections were imported into Cytoscape and were used to construct the network (Fig. 9).

## Discussion

AAA poses a significant threat to human health worldwide due to the risk of ruptured aneurysm. The potential pathophysiological processes involved in the occurrence and the development of AAA have been extensively studied. AAA progression is closely related to the abnormal synthesis and decomposition of the extracellular matrix (ECM), the dysfunction and death of VSMCs and the infiltration of immune cells. The imbalance between the synthesis and degradation of various ECM components causes the anomalous regulation of various cytokines, and ultimately determines the pathophysiological remodeling of the aortic wall (Jana et al., 2019). Thirty percent of VSMCs in AAA are converted into macrophage-like smooth muscle cells, causing abnormal ECM synthesis, elevating a variety of matrix metalloproteinases (MMPs), degrading multiple ECM components and degeneration of the aortic wall. The VSMCs in AAA also undergo apoptosis in response to inflammatory factors (Gurung et al., 2020). Inflammatory cells are also recruited from the lymph nodes outside the vascular wall, the nourishing vessels of the aorta, atherosclerotic plaques and thrombi in the lumen. They infiltrate the entire layer of vessel while secreting various inflammatory factors. These inflammatory cells can also produce a variety of MMPs, ultimately leading to the development of AAA (Li et al., 2018).

However, neither drug experiments nor clinical trials targeting these mechanisms can effectively reduce the AAA occurrence and development. Some studies gradually explored the role of non-coding RNA in AAA. The role of a large number of long non-coding RNAs and miRNAs in AAA were confirmed. However, among circRNAs only circCCDC66, circCBFB and CDR1as were shown to play a role in AAA procession (Kumar et al., 2019; Yang et al., 2020b; Yue et al., 2020; Zhao, Chen & Jiang, 2020). We analyzed four mRNA profiles and one circRNA profile related to AAA in order to better explore the effects of circRNA in AAA. The most important function and pathway of the identified DEGs were associated with the composition and function of the ECM, tumor necrosis factor (TNF)-mediated signaling pathway, and transforming growth factor beta1 (TGF-β1) production after GO function annotation and KEGG pathway enrichment.

During the post-transcriptional regulation of AAA, multiple miRNAs were involved in the above processes: First, the synthesis and decomposition of various proteins and glycans in ECM were affected by miRNA. Han et al. (2020) revealed that the miR-106a expression level in exosomes released from AAA tissues was higher than normal, causing apoptosis and promoting the level of MMP-2 and MMP-12 secreted by VSMCs. These MMPs are the main culprits of ECM degradation. Li et al. (2020) showed that the expression of miR-126a-5p in AAA tissues was significantly reduced. The overexpression of miR-126a-5p improved the degradation of ECM by inhibiting a secreted protease. This process reduced the dilatation of the aorta and improved the survival rate in mice. Other miRNAs, including miR-33, miR-155, miR-516a-5p, miR-205 and miR-145, were shown to play a role in ECM (Chan et al., 2017a; Chan, Cheuk & Cheng, 2017b; Nakao et al., 2017; Wu et al., 2017; Zhang et al., 2018). Second, the TNF-mediated inflammation signaling pathway was found to be closely related to miRNA. Li et al. (2020) found that the expression level of miR-195 in patients with AAA was effectively increased. The suppression of miR-195 inhibited the inflammatory response through the TNF-α/NF-κB and VEGF/PI3K/Akt pathways (Ma et al., 2018). Coincidentally, the expression level of miR-146a in AAA tissue and serum was significantly higher than that of normal controls. Experiments confirmed that miRNA-146a reduced the secretion of TNF-α and blocked the progression of AAA through the CARD10/SIRT1/p65 pathway (Zhang, Wang & Yang, 2020). Finally, the relationship between miRNA and TGF-β was unclear in AAA, but both were shown to play important roles in similar pathophysiological processes. Yang et al. (2020a) determined that miR-26b was under-expressed in Stanford type A aortic dissection (TAAD) patients and that miR-26b impeded TAAD development by regulating TGF-β/Smad3 signaling pathway. They also demonstrated that miR-21 was highly expressed in TAAD and that inhibition of this miRNA could suppress canonical TGF-β signaling. VSMCs that lacked the TGF-β signals tended to switch from a contractile phenotype to a synthetic phenotype, which eventually exacerbated TAAD formation (Huang et al., 2018). In addition, a non-coding RNA sequencing study related to thoracic aortic aneurysms revealed that a variety of miRNAs with significantly altered expression were highly associated with TGF-β (Gasiule et al., 2019).

Ten hub gene-encoded proteins, namely, IL6, RPS27A, JUN, UBC, UBA52, FOS, IL1B, MMP9, SPP1 and CCL2, coupled with a higher degree of connectivity hub were identified after PPI network analysis was conducted. IL6 (interleukin 6) is hyper-expressed in AAA, depending on the context, acting as a multi-faced factor (Kokje et al., 2016). It is protective upon acute injury, but negatively involved in the development of AAA. Nishihara’s group discovered that IL6 plays an important role in continuous cellular infiltration (Nishihara et al., 2017). Liu, Wang & Li (2018) also reported that IL6 encourages inflammation through the NF-κB pathway. However, Kao’s group suggested that IL6 was regarded as a crucial regenerative factor for acute vascular injury (Kao et al., 2018). The hub genes ribosomal protein S27a (RPS27A), ubiquitin C (UBC) and ubiquitin A-52 residue ribosomal protein fusion product 1 (UBA52) encode ubiquitin together with ubiquitin B (UBB). Ubiquitin participates in the formation of the ubiquitin-proteasome system (UPS), which is the main pathway of protein degradation in cells. It regulates embryonic development, cell cycle progression, the immune response, and apoptosis. A study showed that the ubiquitin proteasome system was involved in regulating the differentiation of smooth muscle phenotypes (Paredes et al., 2020), though research on the role of ubiquitination regulation in AAA is quite scarce. In addition, a protein coded by hub gene JUN (Jun proto-oncogene, AP-1 transcription factor subunit) can dimerize with leucine zipper proteins, which is encoded by another hub gene FOS (Fos proto-oncogene, AP-1 transcription factor subunit). Sun et al. (2019) revealed that JUN was involved in the development of AAA and can directly combine with the promoter to mediate the expression of C/EBP homologous protein and induce VSMCs apoptosis and migration. Zhao et al. (2019) revealed that FOS regulated the viability and apoptosis of VSMCs to trigger the progression of AAA. Moreover, as an inflammatory mediator, IL-1B can not only induce the formation of neutrophil extracellular traps in the early stage of AAA formation, but also degrade the extracellular matrix and further lead to the progression of AAA (Jiang et al., 2019; Meher et al., 2018). Furthermore, secreted phosphoprotein 1 (SPP1), also known as osteopontin (OPN), is elevated in the circulating plasma and aortic walls of AAA patients. It leads to an overall pro-inflammatory state and up-regulates the expression of MMP, contributing to the dilation and rupture of the aortic wall (Li et al., 2016; Wang et al., 2018). The hub gene matrix metallopeptidase 9 (MMP9) is critical in AAA (Rabkin, 2017) as it is able to degrade multiple ECM components. The last hub gene, C-C motif chemokine ligand 2 (CCL2), also known as monocyte chemotactic protein 1 (MCP1), displays chemotactic activity for monocytes and basophils. Moehle et al. (2011) determined that this protein contributed to macrophage infiltration into the AAA and acted directly on SMCs to reduce the contractile proteins and induce MMPs.

We found that there were seven intersections between the miRNAs predicted by the Targetscan database and the Circinteractome database after constructing the circRNA-miRNA-mRNA network. Among them, miR-142-5p was verified with the results of previous studies. The other miRNAs: miR-635, miR-527, miR-520h, miR-938, and miR-518a-5p may be affected by circ_0005073 to affect the expression of hub genes such as CCL2, SPP1 and UBA52. The miR-1206 may be affected by circ_0090069 to impact the expression of RPS27A. Previous studies have shown these miRNAs to play a significant role. A study demonstrated that miR-635 could directly interact with circ_0000735. Circ_0000735 functioned as a miR-635 sponge and worked as an important regulator of malignant cancer progression (Tai, Zhang & Liu, 2021). A similar process appeared in miR-527. Circ-CDC45 acted as a sponge and regulated the expression of miR-527 to promote tumor cell growth and invasion (Liu et al., 2020). We also found many relevant studies on miR-520h and its role in diabetes and a variety of tumors have been confirmed. Its downstream targets included toll-like receptor 4, the mechanistic target of rapamycin, phosphatase and tensin homolog, Wnt/β-catenin (Geng et al., 2020; Qi, Yao & Liu, 2020; Wen & Bai, 2021; Zhou et al., 2020b). These targets have been studied in AAA, but the biological role of miR-520h itself and upstream circ_0005073 in AAA has yet to be determined. Similarly, the predicted circ_0090069 downstream microRNA-1206 is one of six annotated miRNAs found in the long noncoding RNA PVT1 locus. It has been shown that PVT1 knockdown inhibited VSMCs apoptosis and ECM disruption in a murine AAA model (Zhang et al., 2019). However, the biological role of circ_0090069 in AAA is unclear.

Our study has several limitations. It is very difficult to obtain a comprehensive description of sample characteristics from the included studies, making it difficult to stratify the information for subgroup analysis to further analyze the factors that affect the results. In addition, the studies of some samples were not successfully published, so it is impossible to clarify the pathological results of all samples. AAA caused by atherosclerosis, cystic degeneration or congenital dysplasia may have slightly different expression profiles.

## Conclusions

Our findings demonstrated that DEGs in AAA may be associated with ECM, TNF-α and TGF-β. These results were consistent with the results of previous studies. After integrated bioinformatics analysis, 10 hub genes and differentially expressed circRNAs were identified from large‑scale samples. Their potential functions in AAA were investigated and we constructed a circRNA-miRNA-mRNA network with linear relation after combining our predictions and previous studies. This network should be further studied for their interaction in AAA progression. Collectively, it is our sincere hope that this study will contribute to the identification of potential therapeutic targets for early intervention in AAA.

## Supplemental Information

10.7717/peerj.12682/supp-1 Supplemental Information 1 The code used to reanalyze the raw data.

10.7717/peerj.12682/supp-2 Supplemental Information 2 R package, R functions and applications used in the code were annotated.

10.7717/peerj.12682/supp-3 Supplemental Information 3 Raw data was processed by batch effect removal.

10.7717/peerj.12682/supp-4 Supplemental Information 4 GO function annotation was performed on the up-regulated DEGs and shown by a bubble plot.

10.7717/peerj.12682/supp-5 Supplemental Information 5 GO function annotation was performed on the down-regulated DEGs and shown by a bubble plot.

10.7717/peerj.12682/supp-6 Supplemental Information 6 KEGG pathway enrichment was performed on the up-regulated DEGs and shown by a bubble plot.

10.7717/peerj.12682/supp-7 Supplemental Information 7 KEGG pathway enrichment was performed on the down-regulated DEGs and shown by a bubble plot.

10.7717/peerj.12682/supp-8 Supplemental Information 8 DEGs between AAA and NOR were dug out.

10.7717/peerj.12682/supp-9 Supplemental Information 9 Details of biological process in GO function annotation of DEGs were listed.

10.7717/peerj.12682/supp-10 Supplemental Information 10 Details of molecular function in GO function annotation of DEGs were listed.

10.7717/peerj.12682/supp-11 Supplemental Information 11 Details of cellular component in GO function annotation of DEGs were listed.

10.7717/peerj.12682/supp-12 Supplemental Information 12 Details of KEGG pathway enrichment of DEGs were listed.

10.7717/peerj.12682/supp-13 Supplemental Information 13 Differentially expressed circRNAs between AAA and NOR were dug out.

10.7717/peerj.12682/supp-14 Supplemental Information 14 Top 10 in PPI ranked by Degree method were listed.

## Additional Information and Declarations

### Data Availability

The following information was supplied regarding data availability: The code that processed the raw data was available in Supplemental Files. The raw data from GEO (GSE47472, GSE52093, GSE57691, GSE98278, and GSE144431) were divided into groups A, B, C, D, and E according to the size of abdominal aortic aneurysm.
