# Supplementary material for: Integrated analysis and the identification of a circRNA-miRNA-mRNA network in the progression of abdominal aortic aneurysm
Source: PeerJ. 2021 Dec 24;9:e12682. doi: 10.7717/peerj.12682 (PMC8711282; doi:10.7717/peerj.12682)
Supplement: Supplemental Information 2 [file peerj-09-12682-s002.docx]

| R functions name | R package | Application |
| --- | --- | --- |
| readIDAT | illuminaio | Analyze the Illumina gene chip data |
| calcNormFactors | edgeR | Calculate the normalization factor |
| voom | Limma | Normalization and correction of batch effects |
| makeContrasts | Limma | Difference analysis |
| ggplot | ggplot2 | Drawing |
| enrichKEGG/ enrichGO | clusterProfiler | Perform enrichment analysis |
