# Supplementary figures and images for: Integrated analysis and the identification of a circRNA-miRNA-mRNA network in the progression of abdominal aortic aneurysm
Source: PeerJ. 2021 Dec 24;9:e12682. doi: 10.7717/peerj.12682 (PMC8711282; doi:10.7717/peerj.12682)

**voom: Mean-variance trend**

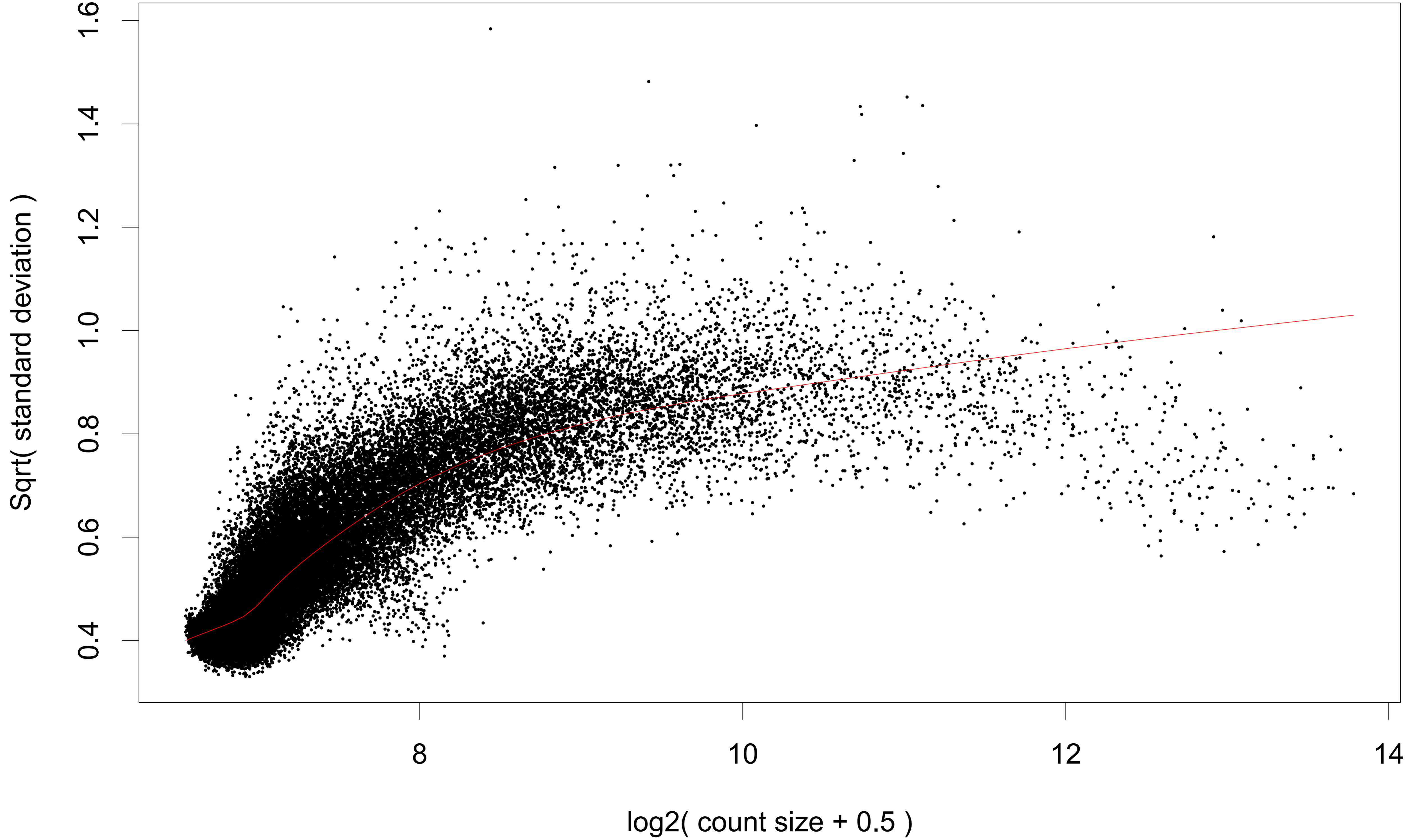

Supplement: Supplemental Information 3 [file peerj-09-12682-s003.pdf]

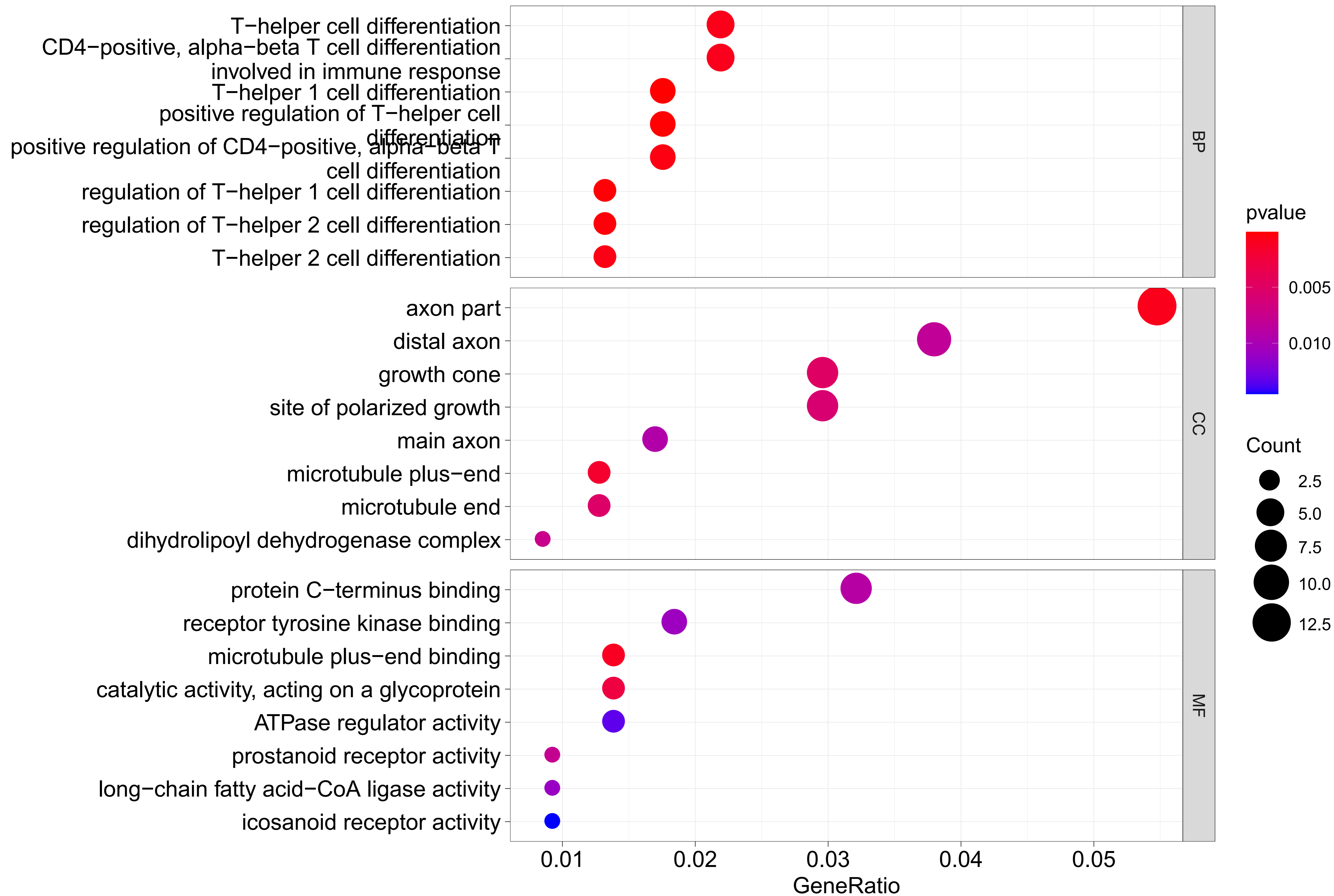

Supplement: Supplemental Information 4 [file peerj-09-12682-s004.pdf]

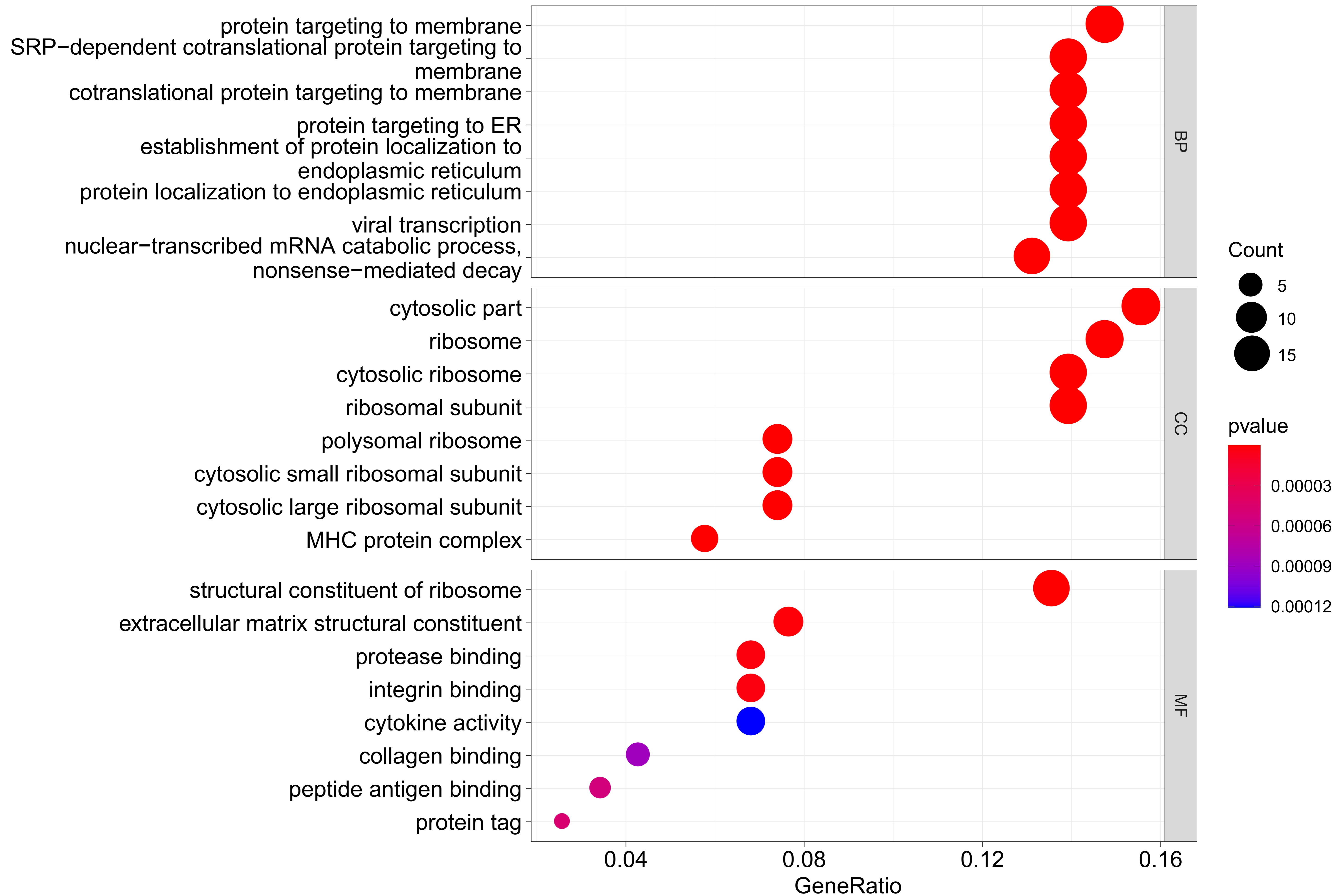

Supplement: Supplemental Information 5 [file peerj-09-12682-s005.pdf]

Glycerophospholipid metabolism

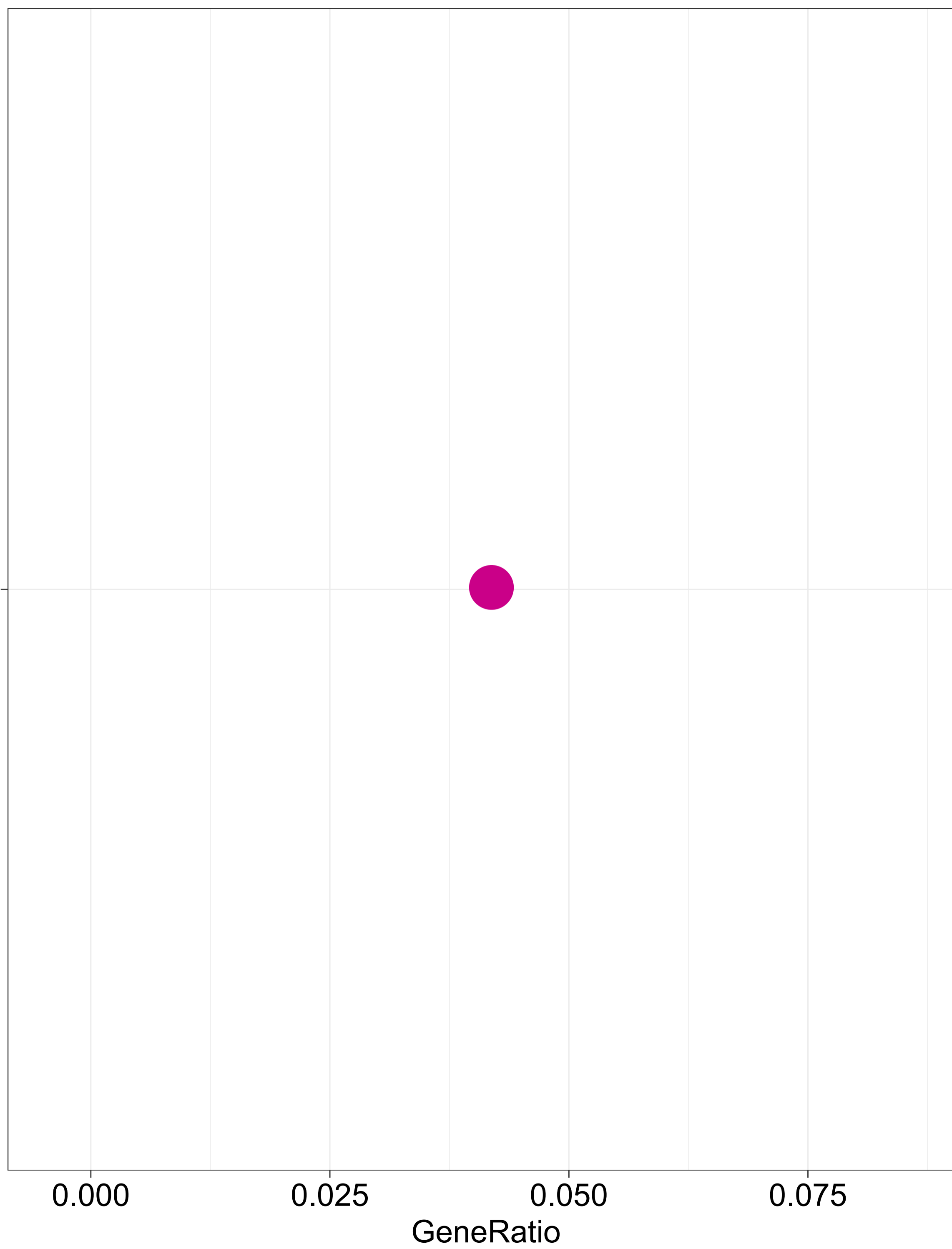

Supplement: Supplemental Information 6 [file peerj-09-12682-s006.pdf]

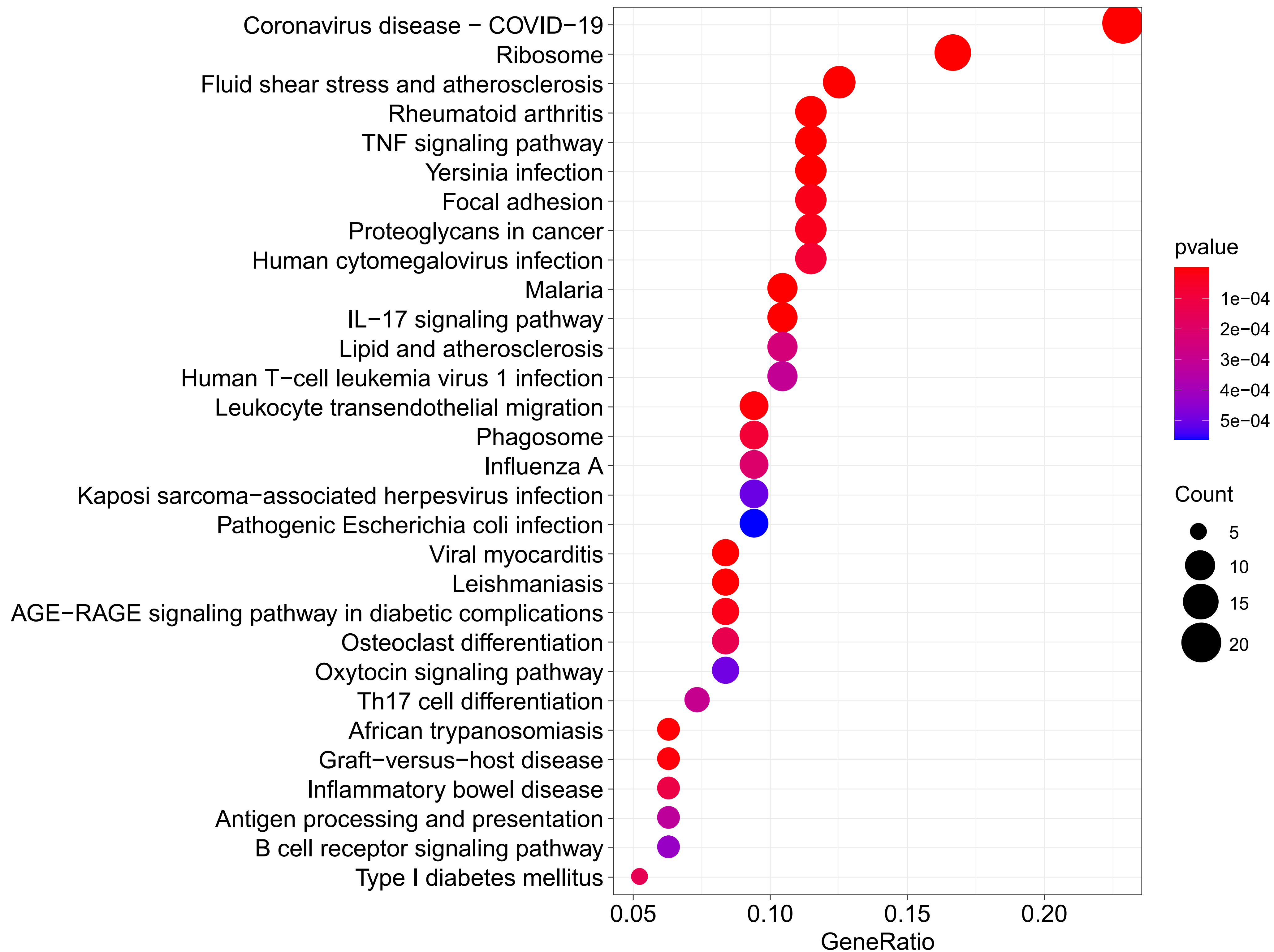

Supplement: Supplemental Information 7 [file peerj-09-12682-s007.pdf]
